# *Listeria monocytogenes* infectious periaortitis: a case report from the infectious disease standpoint

**DOI:** 10.1186/s12879-019-3953-z

**Published:** 2019-04-16

**Authors:** Aurélie Foulex, Matteo Coen, Abdessalam Cherkaoui, Vladimir Lazarevic, Nadia Gaïa, Stefano Leo, Myriam Girard, Damiano Mugnai, Jacques Schrenzel

**Affiliations:** 1 Service of Internal Medicine, Department of Medicine, Geneva University Hospitals, Geneva, Switzerland; 2 Department of Pathology and Immunology, University of Geneva, Faculty of Medicine, Geneva, Switzerland; 3 Bacteriology Laboratory, Department of Genetics and Laboratory Medicine, Geneva University Hospitals, rue Gabrielle Perret-Gentil 4, 1211 Geneva 14, Switzerland; 4 Genomic Research Laboratory, Department of Medical Specialties, Geneva University Hospitals and University of Geneva, CMU–C09.2141, rue Michel Servet 1, 1211 Geneva 4, Switzerland; 5 Service of Cardiac and Vascular Surgery, Department of Surgery, Geneva University Hospitals, rue Gabrielle Perret-Gentil 4, 1211 Geneva 14, Switzerland; 6 Service of Infectious Diseases, Department of Medical Specialties, Geneva University Hospitals, Geneva, Switzerland; 7 Genomic Research Laboratory, CMU–C09.2138, rue Michel Servet 1, 1211 Geneva 4, Switzerland; 8 Bacteriology Laboratory and Service of Infectious Diseases, Department of Medical Specialties, Geneva University Hospitals, rue Gabrielle Perret-Gentil 4, 1211 Geneva 14, Switzerland

**Keywords:** *Listeria monocytogenes*, Metagenomics, Microbiological techniques, Anti-bacterial agents, Endograft infection, Aortic repair, Fastidious organisms, Surgical sampling, Etiologic bacterial diagnosis, Culture-independent methods

## Abstract

**Background:**

Endograft infection is a rare but extremely dangerous complication of aortic repair (25–100% of mortality). We describe here the first case of *Listeria monocytogenes* abdominal periaortitis associated with a vascular graft. We also discuss the differential diagnosis of periaortitis and provide a literature review of *L. monocytogenes* infectious aortitis.

**Case presentation:**

Nine months after endovascular treatment of an abdominal aortic aneurysm (abdominal stent graft), a 76-year-old man was admitted for severe abdominal pain radiating to the back. Laboratory tests were normal apart from elevated C-reactive protein (CRP). Injected abdominal computed tomography (CT) showed infiltration of the fat tissues around the aortic endoprosthesis and aneurysmal sac expansion; positron emission tomography with 2-deoxy-2-[fluorine-18]fluoro- D-glucose integrated with computed tomography (18F-FDG PET/CT) showed a hypermetabolic mass in contact with the endoprosthesis. Blood cultures were negative. At surgical revision, an infra-renal peri-aortic abscess was evident; post-operative antibiotic therapy with ciprofloxacin and doxycycline was started. Cultures of intraoperative samples were positive for *L. monocytogenes*. Results were further confirmed by a broad-range polymerase chain reaction (PCR) and next-generation sequencing. Antibiotic treatment was switched to intravenous amoxicillin for 6 weeks. Evolution was uneventful with decrease of inflammatory parameters and regression of the abscess.

**Conclusion:**

An etiologic bacterial diagnosis before starting antibiotic therapy is paramount; nevertheless, culture-independent methods may provide a microbiological diagnosis in those cases where antimicrobials are empirically used and when cultures remain negative.

## Background

Aortic inflammatory disease is rare (1.38 cases/100,000 inhabitants) and prevails in middle aged (50–60 years old) men (male/female ratio 2:1-3:1). It comprises two main entities: aortitis and periaortitis, both characterised by the infiltration of the arterial layer by inflammatory cells and the disruption of the arterial structure [1]. Nevertheless, while aortitis is confined to the arterial wall, in (chronic) periaortitis adventitial inflammation develops and leads to the involvement of adjacent retroperitoneal structures (e.g. ureters and inferior vena cava). Thoracic involvement has been described, despite the fact that this infection typically affects the infrarenal aorta and/or the iliac arteries.

Chronic periaortitis is classically considered to encompass three distinct entities: idiopathic retroperitoneal fibrosis, inflammatory abdominal aortic aneurysms and perianeurysmal retroperitoneal fibrosis [2]. Clinically indistinguishable (i.e. dull abdominal pain, fever, fatigue, weight loss) and histologically similar (i.e. a thick fibro-inflammatory infiltrate rich in lymphocytes and collagen), some authors hypothesize a spectrum of the same disease [3]. Once considered a local complication of aortic atherosclerosis, chronic periaortitis seems rather to be a manifestation of a systemic disease (e.g. ankylosing spondylitis, rheumatoid arthritis, IgG4-related disease, Wegener’s granulomatosis, polyarteritis nodosa) [4]. If an underlying immune disease is excluded, the clinician should actively investigate for more unusual causes, like tumors, drugs, radiotherapy, trauma, major abdominal surgery, myeloproliferative disorders (Erdheim-Chester disease), exposure to toxics (e.g. asbestos) and drugs (e.g. ergot-derived drugs) as well as infections [5].

Before the beginning of the “antibiotic era”, the frequency of aortic infections ranged from 2.6 to 3.4% [6]. Although aortic infections are nowadays much rarer, they remain life-threatening. Antibiotics, indeed, lead to the near complete disappearance of tuberculous and syphilitic aortitis, as well as a drastic reduction in endocarditis-associated septic embolization and mycotic aneurysms (“mycotic” is misnomer: Osler used this term to describe the “fresh fungous” appearance of these bacterial lesions) [7].

Until the intimal layer is intact, the aorta is extremely resistant to infections. Therefore, haematogenous infections often arise in the context of a damaged endothelium, like in atherosclerotic lesions, aneurysms, aortitis, and congenital abnormalities. Besides bacteraemia, which is the most likely route of infection, septic embolization to the vasa vasorum (as in infectious endocarditis), extension form a neighbouring infectious focus as well as contamination in the setting of intravenous drug use, trauma and invasive intravascular procedures have been described [8].

Infectious periaortitis seems to occur even less frequently than aortitis, [9] with only one possible case appearing in medical literature [10]. We believe that insufficient or unreliable diagnostic techniques are likely responsible for an underestimation of this condition.

From the infectious diseases standpoint, clinicians should consider the possibility of infections sustained by fastidious organisms commonly missed by conventional cultures, e.g. *Brucella* spp. and *L. monocytogenes*.

In this paper, we describe the first reported case of *L. monocytogenes* abdominal periaortitis. Moreover, we provide a review of the literature and we discuss about a culture-independent method for microbiological diagnosis.

## Case presentation

A 76-year-old man with a history of chronic obstructive pulmonary disease (Gold grade 4) and type 2 diabetes was admitted for abdominal pain radiating to the back for 10 days; pain was punctuated by brief episodes of diarrhoea. Nine months before admission, the patient had undergone endovascular treatment of an abdominal aortic aneurysm (abdominal stent graft).

Patient was afebrile, and physical examination was unremarkable apart from diffuse abdominal tenderness without guarding or rebound. Laboratory tests showed mild anaemia (111 g/L), a total white blood cell count of 7.6  ×  10^9^/l with a neutrophil count of 5.21  ×  10^9^/l; CRP was 233.5 mg/l and procalcitonin 0.09 μg/L. Kidney (creatinine, urea, sodium and potassium dosage) and liver (ALT, SGOT, SGPT, bilirubin, gamma-glutamyltranspeptidase dosage) function tests were within normal ranges.

**Fig. 1:**
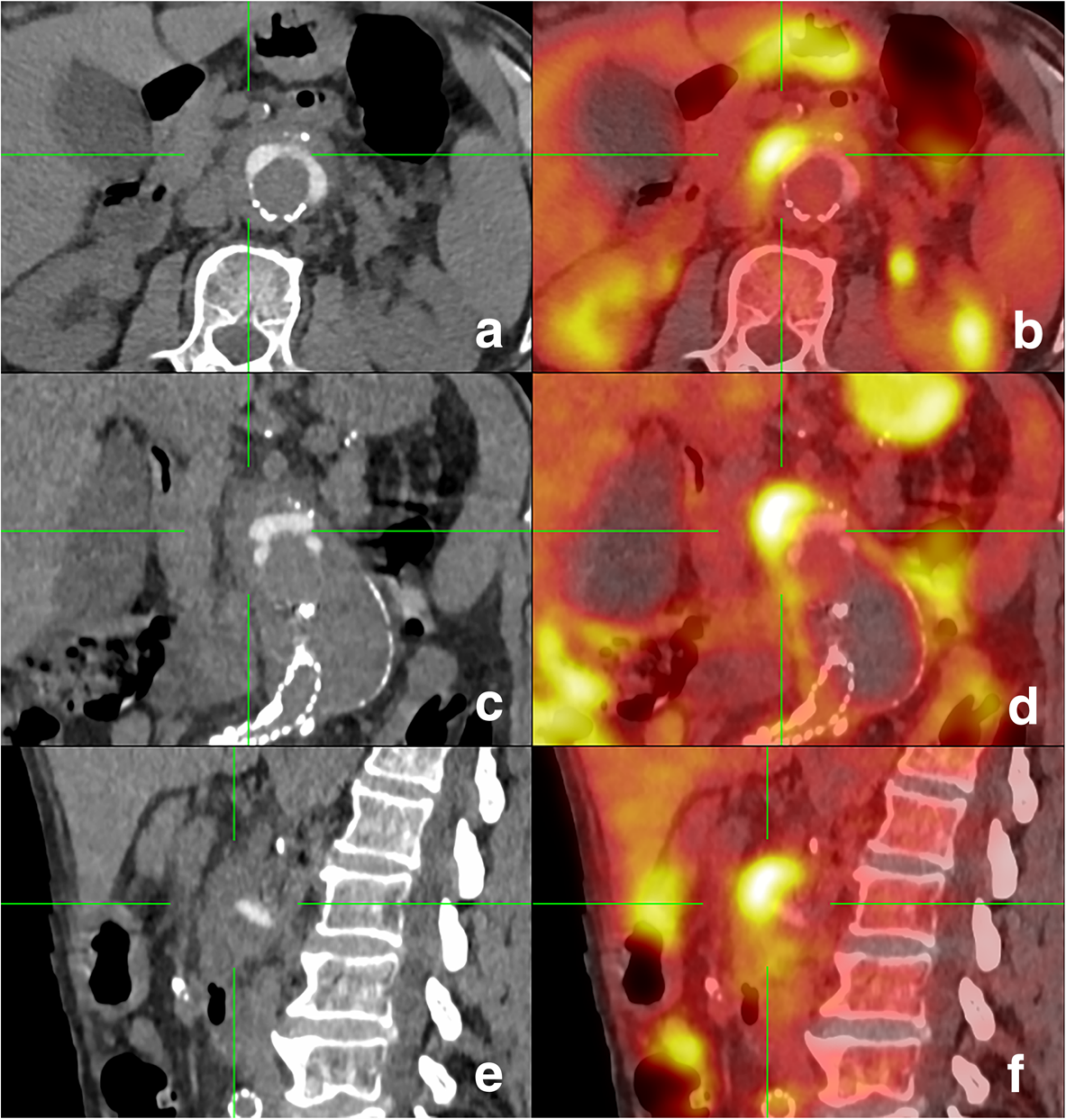
^18^F-FDG PET/CT, axial (**a**-**b**), coronal (**c**-**d**) and sagittal (**e**-**f**) planes. CT shows infiltration of the fat tissues around the aortic endoprosthesis (**a**, **c**, **e**); corresponding PET (**b**, **d**, **f**) reveals an intense ^18^F-FDG hypercaptating mass in contact with the superior antero-medial of the endoprosthesis

A contrast-enhanced abdominal computed tomography (CT) scan showed infiltration of the fatty tissues around the aortic endoprosthesis and increase of the aneurysmal sac expansion by 6.0 mm (57 versus 51 mm) compared to a previous CT performed 2 months earlier; no endoleak was observed. No other abnormalities were observed. ^18^F-FDG PET/CT (Fig. 1, a-e) showed a hypermetabolic (SUVmax = 8.5) mass with a diameter of 15 mm in contact with the superior antero-medial of the endoprosthesis, suggesting an abscess. CT colonography excluded neoplasia.

Serum immunofixation was normal, without increase in the IgG4 subclass. The interferon-gamma reactivitiy assay was negative. Serologies for *Treponema pallidum*, *Bartonella henselae* and *Coxiella burnetii* were negative, as were *Brucella* agglutination tests (Rose-Bengal and Wright). Multiple blood cultures showed no growth. Real-Time Quantitative broad-range PCR on blood was negative. Stool PCR testing for *Campylobacter jejuni/coli*, *Shigella* spp. and *Salmonella* spp. was negative.

Multidisciplinary consensus involving interventional radiologists, vascular surgeons, infectious disease specialists, and internists, led to the decision for a surgical revision. During the procedure, an infrarenal peri-aortic abscess was noted, from which samples were performed. In the post-operative period and before culture results were available, patient was started on oral ciprofloxacin (500 mg/12 h) and doxycyclin (100 mg/12 h). Histopathological examination of the intraoperative specimens revealed fibrous tissue with accompanying inflammation rich in histiocytes and cholesterol crystals; no microorganisms were identified on hematoxylin/eosin, Gram or Grocott stains.

Cultures of intraoperative samples (5/5) became positive for *L. monocytogenes* after 10 days incubation. These results were further confirmed by a broad-range PCR. Susceptibility testing (Kirby-Bauer disk diffusion susceptibility tests (SirScan 200 Automatic, i2A, France) interpreted according to the EUCAST clinical MIC breakpoints) [11] showed a pan-susceptible (“wild type”) *L. monocytogenes* strain with a minimum inhibitory concentration [MIC] for amoxicillin of 0.380 mg/l. Antibiotic treatment was then switched to intravenous amoxicillin ((2 g/6 h) for a duration of 6 weeks. The patient’s clinical course was favourable, and CRP levels became within normal range. At the end of antibiotic course, an abdominal CT scan showed regression of the peri-aortic abscess and collection. At 6 months follow-up the patient was well, without recurrence.

### DNA extraction

Frozen aortic tissue was cut into small pieces on a disposable Petri dish support using a scalpel. DNA was extracted from 83 mg of shredded sample using the Ultra-Deep Microbiome Prep kit (Molzym, Bremen, Germany) for enrichment of bacteria/fungal DNA, according to the manufacturer’s instructions (Version 2.0) for tissue samples.

### Quantitative PCR (qPCR) assays

The concentration of bacterial and human DNA was determined by qPCR experiments as previously described [12], using 16S rRNA and beta-actin reference genes, respectively. The reference curves for bacterial and human DNA quantitation were generated using known concentrations of *Escherichia coli* DH5α genomic DNA and human genomic DNA from the TaqMan beta-Actin Detection Reagent kit (Applied Biosystems, Framingham, MA), respectively.

### DNA sequencing

Metagenomic libraries were prepared from 1 ng DNA (sum of bacterial and human DNA load determined by qPCR), using Nextera XT DNA Sample Preparation Kit according to Illumina (San Diego, USA) instructions, except that 16 (instead of 12) PCR enrichment cycles were used. The libraries were sequenced for 2 × 250 + 8 cycles on an Illumina MiSeq instrument at Fasteris (Plan-les-Ouates, Switzerland) using the MiSeq Reagent Kit v3 and MiSeq Control Software 2.6.2.1.

### Bioinformatics analysis

The Trimmomatic package was used by the sequencing service provider to remove bases that correspond to the standard Illumina adapters and to trim low-quality ends of reads at the beginning of a 4-base wide sliding window with an average Phred quality < 5 [13]. We further trimmed the reads with the low-quality base score of Q10 and a mean quality score Q30 over a sliding 20-base window. Any read that, after trimming, had a length < 150 bases was discarded. To filter out putative artificial replicate reads, we used a home-made script which retains only the longest sequence from those with identical first 100 bases in either forward or reverse reads. Unpaired forward or reverse reads were discarded. Remaining reads were classified with Kraken v.0.10.5-beta with the default parameters [14]. After filtering out the read pairs that matched human genome, the data were deposited to European Nucleotide Archive (ENA) database (accession number PRJEB21816).

**Fig. 2:**
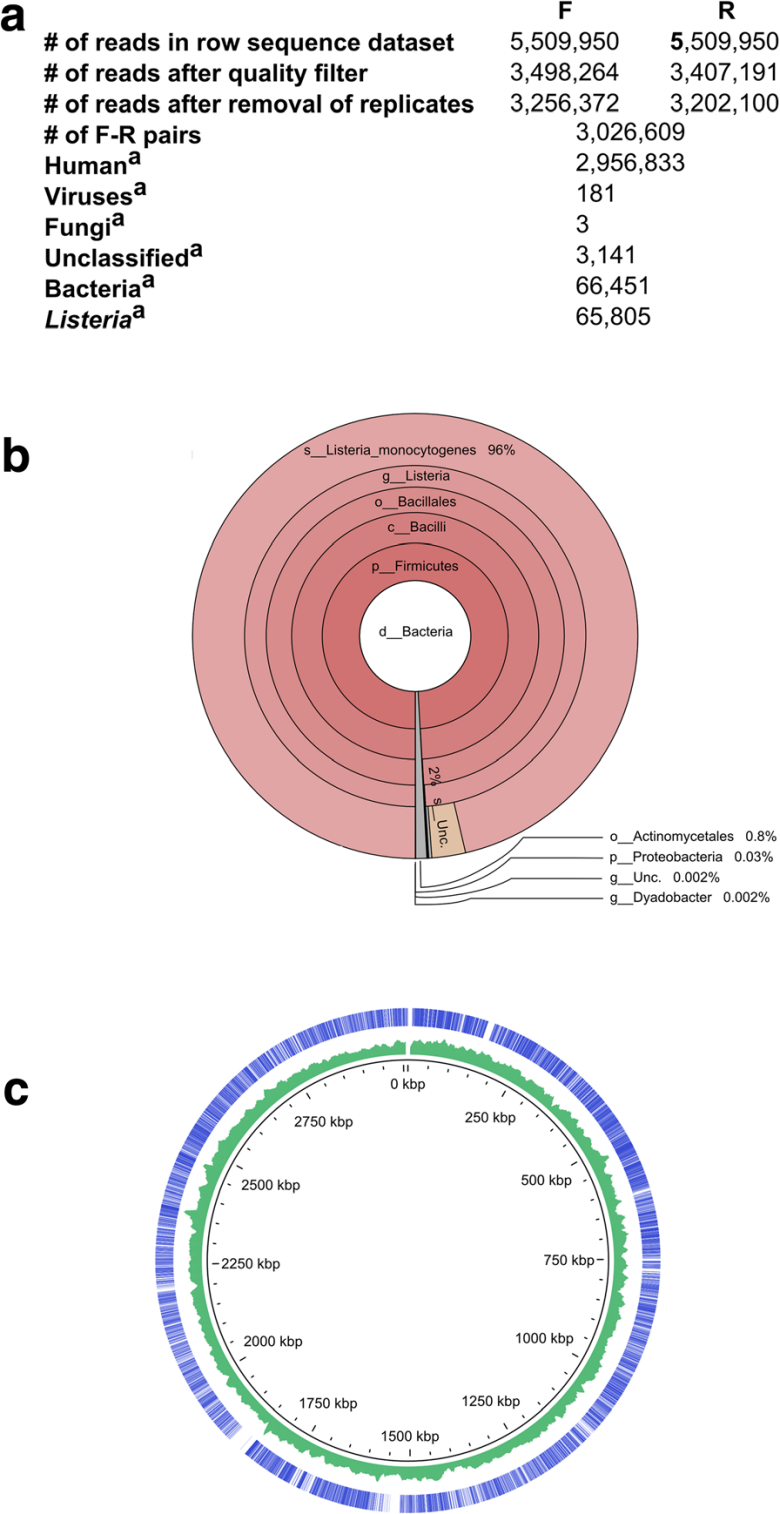
Number and taxonomic assignments of sequence reads obtained by WMGS of DNA extracts from the perioaortic tissue sample. F, forwards reads; R, reverse reads. **a**. Number of sequence reads at different steps of data analysis. **b**. Krona plots of bacterial taxa identified by Kraken [34]. **c**. Ring diagram showing the genome of the *L. monocytogenes* strain 6179 chromosome sequence (GenBank accession number NZ_HG813249). Outer circle depicts the chromosome regions to which mapped pairs of sequence reads indicated by bars. Middle circle shows G + C content expressed as percentage. The inner ring indicates chromosomal position. The figure was generated using GView [17]

The reads classified by Kraken to genus *Listeria* were retrieved and mapped to complete reference genomic sequences of *Listeria* strains (downloaded from the NCBI Reference Sequence (RefSeq) prokaryotic genome collection) [15] using USEARCH 8.1.11861 [16] (−ublast -id 0.98 -cov 0.98 -top_hits_only -strand both -evalue 0.00001). The number of hits was counted for each *Listeria* strain. The strain with the maximum number of hits was retained as a reference for read mapping using GView [17]. MetaPhlAn2 taxonomic profiling, based on read mapping against ~ 1 million markers from more than 7500 species was used with default settings [18] (Fig. 2).

Although culture and broad-range PCR detected *L. monocytogenes* in the clinical specimen, we performed next-generation sequencing in order to determine the clinical utility of such a technique. We extracted DNA from the aortic tissue using a protocol that improves the bacterial-to-human DNA ratio based on selective lysis of host cells and removal of host DNA before the lysis of bacterial cells [19, 20]. The concentrations of human and bacterial DNA in purified extracts were 5983 and 214 pg/μL, respectively, as determined by qPCR assays. The whole metagenome shotgun sequencing generated 5,509,950 raw paired reads of which 3,026,609 passed quality control (Fig. 1a). In line with qPCR results, most reads (97.7%) corresponded to human genome sequence. Of 66,451 read pairs classified by Kraken as bacterial, 65,805 (99%) were assigned to the genus *Listeria* and 64,035 (96.4%) to *L. monocytogenes* (Fig. 1 b, c). Most of the remaining bacterial reads corresponded to *Propionibacterium* (464 reads) and *Micrococcus* (32 reads), both of which are frequent reagent contaminants. We have previously identified *Propionibacterium* in negative extraction controls (PRJEB20595) obtained with a DNA extraction kit of the same lot [20]. Mapping of sequencing reads against clade-specific marker genes using MetaPhlAn2 confirmed the results obtained by Kraken. The MetaPhlAn2 taxonomic profiling showed high abundance of *Listeria* (99.91%) and *L. monocytogenes* (96.28%), with a minor presence of *Cutibacterium acnes* (formerly *Propionibacterium acnes*) (0.089%).

## Discussion and conclusions

Endograft infection after aortic repair can spread to the arterial wall and disrupt it, causing graft migration, aortic dilatation and rupture. This condition is rare, occurring between < 1 and 3% following endovascular or open procedures to the thoracic aorta, and between 0.4 and 1.3% following endovascular or open abdominal aortic repair. Mortality is high 25–100%, with higher rates when a conservative strategy is adopted [21, 22].

Whereas *Staphylococci* and *Salmonella* spp*.* (in particular *Salmonella enterica* serovar Typhimurium, Enteritidis and Cholerasuis) are the most common microorganisms isolated in native aorta infections, accounting for approximately 50–60% and 30–40% of all cases respectively, Staphylococci, *E.coli* and *Pseudomonas aeruginosa* predominate in endograft infections [23]. Clinicians should consider the possibility of fastidious organisms commonly missed by conventional cultures (e.g. *Brucella* spp*.* and *L. monocytogenes*). Bona fide mycotic aortitis and endograft infection have been rarely reported [24].

To date only 18 cases of infectious aortitis due to *L. monocytogenes* have been described. Of these, 2 occurred in the context of thoracic aortic aneurysms [25, 26], 9 in abdominal aortic aneurysms [27–29] and 7 in endovascular aortic graft [30–33]. Our case is, to the best of our knowledge, the first case of *L. monocytogenes* abdominal periaortitis associated with a vascular graft.

In this case, diagnosis was made only through surgical sampling (blood cultures were all negative, despite absence of any antimicrobial therapy). We strongly emphasize the need to establish an etiologic bacterial diagnosis by avoiding empiric and precipitated use of antimicrobials, as evidenced here. Unbiased, culture-independent methods could help elucidating cases where antimicrobials are empirically used, nevertheless these methods are often non-affordable nor fully evaluated yet. Standard, state-of-the-art diagnostic methods are still based on bacterial culture (on conventional culture media) performed before initiation of antibiotics.

Given this quite unexpected result and the high risk of false-negative culture results, if antibiotics had been administered prior to surgery, we wonder whether an unbiased, culture-independent method could have provided this microbiological diagnosis.

## Abbreviations

18F-FDG PET/CT: Positron emission tomography with 2-deoxy-2-[fluorine-18]fluoro- D-glucose integrated with computed tomography
CRP: C-reactive protein
CT: Computed tomography
MIC: Minimum inhibitory concentration
PCR: Polymerase chain reaction
qPCR: Quantitative polymerase chain reaction
